# Validation of aerobic threshold assessment using a sweat lactate sensor during arm crank exercise by healthy adults

**DOI:** 10.14814/phy2.71002

**Published:** 2026-07-06

**Authors:** Tomonori Sawada, Hiroki Okawara, Kazuki Minami, Shintaro Narushima, Ayaka Shiratori, Yoshinori Katsumata, Masaya Nakamura, Takeo Nagura, Daisuke Nakashima

**Affiliations:** ^1^ Department of Orthopaedic Surgery Keio University School of Medicine Shinjuku‐ku Tokyo Japan; ^2^ Institute for Integrated Sports Medicine Keio University School of Medicine Shinjuku‐ku Tokyo Japan; ^3^ Department of Cardiology Keio University School of Medicine Shinjuku‐ku Tokyo Japan; ^4^ Department of Clinical Biomechanics Keio University School of Medicine Shinjuku‐ku Tokyo Japan

**Keywords:** aerobic threshold, arm crank ergometer, sweat lactate sensor, sweat rate, ventilatory threshold

## Abstract

Assessment of the aerobic threshold (AeT) using a sweat lactate sensor has been validated primarily during lower limb exercise. This study aimed to examine whether sweat‐based AeT assessment is also applicable during upper limb exercise. In this cross‐sectional study, 27 healthy young individuals performed an incremental arm crank ergometer test while sweat lactate levels were continuously monitored using a wearable sensor attached to the forehead. Expired gas variables, sweat rate, and heart rate were simultaneously recorded. Correlation analyses were conducted between the lactate threshold in sweat (sLT) and the ventilatory threshold (VT1). Sweating was observed in all participants, and the sLT was successfully identified using the sensor. Strong correlations were observed between sLT and VT1 (workload: *r* = 0.904, exercise time: *r* = 0.859, both *p* < 0.01). Furthermore, no significant differences were observed between the heart rates at sLT and VT1. These findings demonstrate that sLT corresponds closely with VT1 during arm crank exercise, supporting the robustness of sweat‐based AeT assessment across different exercise modalities.

## 1 INTRODUCTION

Exercise intensity domains are commonly delineated by physiological breakpoints that reflect transitions in metabolic regulation. Among these, the aerobic threshold (AeT) represents the first systematic shift from predominantly oxidative metabolism toward increasing anaerobic contribution, accompanied by a rise in lactate production and associated metabolic acidosis (Wasserman, 1984; Wasserman et al., 1973; Wasserman & McIlroy, 1964). Identification of this threshold is fundamental for exercise prescription in clinical practice and scientific research (Binder et al., 2008; Mann et al., 2013).

Traditionally, AeT has been estimated using either ventilatory or blood lactate indices (Faude et al., 2009; Hollmann, 2001; Sales et al., 2019). The ventilatory threshold (VT) is derived from nonlinear changes in pulmonary gas exchange variables, with VT1 representing the first breakpoint identified by the V‐slope method (Beaver et al., 1986) and VT2 corresponding to the respiratory compensation point (Whipp et al., 1981). Similarly, the lactate threshold (LT) is determined from characteristic elevations in blood lactate concentration, with LT1 indicating the initial sustained rise above resting levels (Beaver et al., 1985) and LT2 reflecting a more rapid accumulation at higher intensities (Jamnick et al., 2020). These breakpoints have been conceptually unified within a two‐threshold framework describing aerobic and anaerobic transition domains (Skinner & McLellan, 1980). Although ventilatory and lactate markers arise from distinct physiological systems, VT1 and LT1 are generally considered to represent comparable metabolic transitions (MacIntosh et al., 2021; Tanner et al., 2024). Despite their physiological validity, both methods present practical limitations: gas exchange analysis requires specialized laboratory equipment and technical expertise, whereas blood lactate assessment necessitates repeated invasive sampling and may be influenced by the duration of exercise stages because of lactate kinetics. Such constraints limit the feasibility of threshold‐based exercise prescription in many real‐world and community settings.

Recent advances in wearable biosensing technologies have opened new possibilities for non‐invasive metabolic monitoring during exercise. Sweat has attracted attention as an alternative biological matrix for lactate detection, offering the advantages of continuous sampling and minimal invasiveness (Van Hoovels et al., 2021; Yang et al., 2024). Using a compact wearable sensor capable of real‐time sweat lactate monitoring, our research group and others have demonstrated that a sweat lactate threshold (sLT) can be identified during incremental lower limb exercise and that this breakpoint corresponds primarily to VT1 or LT1 (Katsumata et al., 2024; Maeda et al., 2023; Muramoto et al., 2023; Okawara et al., 2023; Sawada et al., 2026; Seki et al., 2021). These findings suggest that sweat lactate dynamics may capture systemic metabolic transitions and thus serve as a practical surrogate for conventional threshold assessment. However, validation studies to date have largely been confined to lower limb exercise, typically using cycling. Upper limb exercise performed with an arm crank ergometer is characterized by lower peak oxygen uptake and earlier attainment of the anaerobic threshold compared with cycling (Davis et al., 1976; Orr et al., 2013; Reybrouck et al., 1975). Because arm exercise generally elicits lower absolute workloads and reduced whole‐body metabolic demand, sweating responses may also differ from those observed during lower limb exercise. Given that the identification of sLT depends on detectable changes in sweat lactate dynamics during incremental exercise, such differences could influence the timing or clarity of the sweat lactate breakpoint. Therefore, whether the close correspondence between sLT and VT1 observed during lower limb exercise can be generalized to upper limb exercise remains uncertain.

Accordingly, this study aimed to validate the assessment of AeT using sweat lactate during an incremental exercise test performed with an arm crank ergometer. We hypothesized that the sweat lactate threshold identified during upper limb exercise would closely align with VT1 derived from expired gas analysis, thereby supporting the robustness of sweat‐based AeT estimation across different exercise modalities.

## 2 METHODS

### 2.1 Study design and ethical approval

This cross‐sectional validation study was conducted in accordance with the Declaration of Helsinki and the ethical guidelines for medical and health research involving human participants. The study protocol was approved by the Institutional Review Board of Keio University School of Medicine (approval number: 20180357). Written informed consent was obtained from all participants prior to their enrolment.

### 2.2 Participants

Sample size estimation was conducted a priori, based on an anticipated correlation coefficient of 0.6 or higher (Katsumata et al., 2024), with a two‐tailed α level of 0.05 and a statistical power (1 ‐ β) of 0.9, resulting in a minimum of 24 participants. To account for potential missing data or participant attrition, the sample size for this study was increased to 27. Recruitment of participants occurred between September 2024 and November 2024. The study included 27 healthy male and female participants, all of whom were free from musculoskeletal injuries or cardiorespiratory diseases. Healthy adults of varying fitness levels, regardless of exercise habits, were recruited primarily from a single university.

### 2.3 Experimental protocol

**FIGURE 1:**
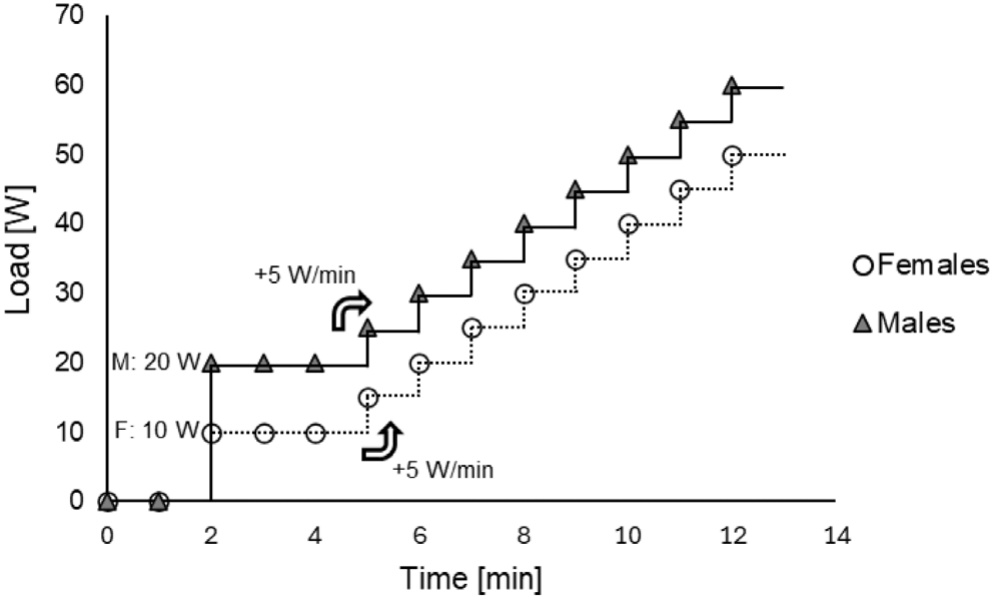
Experimental protocol.

All testing was conducted in a temperature‐controlled laboratory (22°C–24°C) to ensure experiment consistency. Given that alcohol and caffeine can influence lactate metabolism during exercise (Abbotts et al., 2023; Dos Santos et al., 2024), participants were instructed to abstain from alcohol or caffeine for 12 h prior to testing (Maeda et al., 2023). Participants were also required to be well‐hydrated and to avoid vigorous exercise for 3 h before the exercise test. Incremental exercise was performed using a manually braked arm crank ergometer (881E Rehab Trainer; Monark Exercise AB, Vansbro, Sweden). Following a 3‐min warm‐up (20 W for male and 10 W for female), the workload was increased using a step incremental protocol with 5 W increments every minute (Figure 1), while pedaling cadence was maintained at 50 rpm. The test concluded upon volitional exhaustion or the inability to sustain the prescribed cadence. During exercise, sweat lactate levels were monitored using a sweat lactate sensor (Grace Imaging Inc., Tokyo, Japan) attached to the forehead, which was selected because of its relatively high sweat production and stable sensor attachment during exercise. Because the sensor exhibits a transient increase in electrical current value immediately after skin contact, measurements were initiated only after visually confirming that the electrical current value had decreased and reached a stable plateau. The local sweat rate (mg cm^−2^ min^−1^) was measured at a sampling rate of 1 Hz in the same area using a perspiration meter (SKN‐2000 M; SKINOS Co., Ltd., Ueda, Japan). Additionally, heart rate (HR) was monitored using a Polar H10 device (Polar Electro Japan Co., Ltd., Tokyo, Japan).

### 2.4 LT in sweat measurement

The sweat lactate was quantified using a wearable sensor that measured lactate concentration as an electrical current, generated through its interaction with sweat lactate (Seki et al., 2021). The sensor chip contains lactate oxidase, which reacts with lactate in sweat to generate pyruvate and H_2_O_2_, resulting in an electrical current proportional to sweat lactate levels. Therefore, sweat lactate values were expressed as electrical current (μA) rather than as absolute lactate concentrations (mmol/L). This current was continuously recorded, ranging from 0.1 to 80 μA, with increments of 0.1 μA. The sensor exhibited a linear amperometric response to lactate concentrations within the physiological range of 0–10 mmol/L and demonstrated particularly high sensitivity (2.4 A/mM) in the 0–5 mmol/L range (Seki et al., 2021). This range is physiologically relevant for LT1/VT1, as lactate concentrations around 2 mmol/L have been associated with these thresholds (Binder et al., 2008). The sensor also demonstrated reproducible amperometric responses during repeated exposure to the same lactate concentration, supporting its analytical reliability and stability (Seki et al., 2021). Additionally, under simulated sweat conditions, the sensor maintained robust responses to lactate despite variations in pH (5, 6, 7, and 8), temperature (25, 31, and 36°C), and electrolyte concentrations (NaCl and KCl), supporting its robustness and applicability in real‐world sweating scenarios (Muramoto et al., 2023). Data were collected at a sampling rate of 1 Hz for mobile applications via Bluetooth connection. The recorded data were converted into moving average values over 13‐s intervals. The sLT was identified as the first significant rise in a graphical plot of sweat lactate from baseline, which correlated well with VT1 or LT1 determined from blood lactate concentration (Katsumata et al., 2024; Maeda et al., 2023; Muramoto et al., 2023; Okawara et al., 2023, 2024; Sawada et al., 2026; Seki et al., 2021). Subsequently, the sLT was evaluated by three researchers in consultation (Seki et al., 2021).

### 2.5 Respiratory gas analysis and VT

The expired gas flow through the mask was measured using an automated breath‐by‐breath system (Aeromonitor®; Minato Medical Science Co., Ltd., Osaka, Japan). A three‐way calibration process was conducted for the flow‐volume sensor analyzer and delay‐time calibration. Parameters of respiratory gas exchange, including minute ventilation (VE), oxygen consumption (VO_2_), and carbon dioxide production (VCO2), were continuously monitored and averaged every 10 s. VT1 was determined using a combination of the ventilatory equivalent and V‐slope methods (Amann et al., 2006; Binder et al., 2008; Gaskill et al., 2001) with the aid of manual operating software. A researcher who was independent from the three investigators involved in the sLT determination analyzed the respiratory gas exchange data and determined VT1, thereby minimizing potential assessment bias between the two threshold measurements.

### 2.6 Statistical analyses

Data are presented as mean ± standard deviation (SD). The relationship between exercise time and work rate at sLT and VT1 was investigated using Pearson's product–moment correlation coefficient. In addition, Bland–Altman analysis was performed to assess agreement between the thresholds (Bland & Altman, 1986). Fixed and proportional bias, both forms of systematic bias, were evaluated. Fixed bias was assessed by examining whether the mean difference and its 95% confidence interval included zero, whereas proportional bias was assessed by linear regression analysis between the differences and the means, with the slope tested against zero. Furthermore, to compare heart rate, VO_2_, sweat rate, and sweat lactate levels during the arm crank exercise test across predefined exercise stages (warm‐up onset, end of warm‐up, sLT, VT1, and end of exercise), repeated‐measures analysis of variance (ANOVA) with Bonferroni adjusted post hoc testing was applied. All analyses were performed using SPSS Statistics version 29.0 (IBM Corp., Armonk, NY, USA), with statistical significance set at *p* < 0.05.

## 3 RESULTS

**FIGURE 2:**
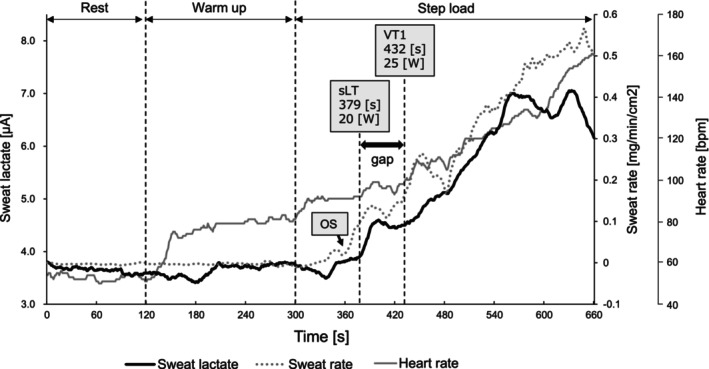
Representative graphs of sweat lactate levels (black solid line), local sweat rate (gray dotted line), and heart rate (gray solid line) with a step incremental protocol (5 W min^−1^) arm crank ergometer. OS: Onset of sweating, sLT: Sweat lactate threshold, VT1: Ventilatory threshold.

The baseline characteristics of the participants are summarized in Table 1. The mean room temperature and humidity were 22.6°C ± 1.0°C and 54.9% ± 7.8%, respectively. Figure 2 presents a representative example of the sweat lactate, sweat rate, and heart rate during the exercise test. The sLT was detectable in all participants at the point where sweat lactate began to increase. Although the sweat lactate and sweat rate increased with increasing exercise load, the increase in sweat rate (i.e., onset of sweating) generally preceded the sLT and was not always associated with the onset of sweating. The time to reach sLT and the onset of sweating were 374.9 ± 95.3 s and 346.5 ± 111.3 s, respectively.

**TABLE 1:** Participant characteristics (*n* = 27).

Sex	Males: 9, females: 18
Age (years)	20.9 ± 1.1
Height (cm)	164.6 ± 8.0
Body mass (kg)	57.3 ± 8.6
Body mass index (kg m^−2^)	21.1 ± 2.0
Average frequency of exercise per week	2.6 ± 1.9
Average exercise time per day (h)	1.8 ± 1.1

*Note*: Data are presented as the mean ± standard deviation.

**FIGURE 3:**
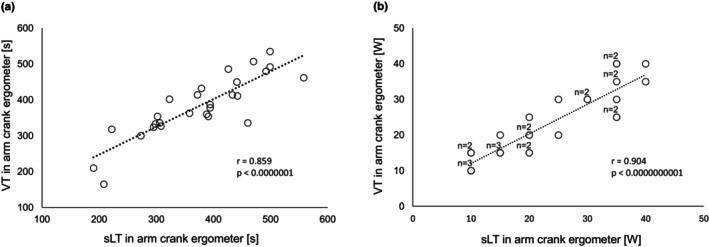
Relationship between sLT and VT1. (a) sLT vs. VT1 (s). (b) sLT vs. VT1 (w). sLT: Sweat lactate threshold, VT1: Ventilatory threshold.

**FIGURE 4:**
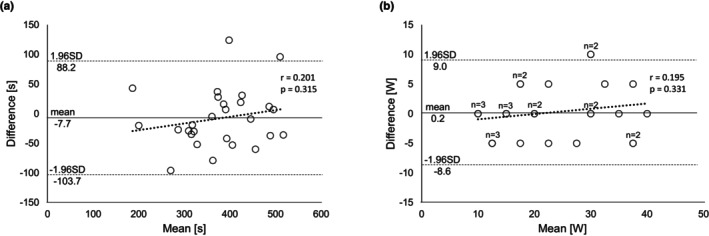
Bland–Altman plot showing the respective differences between sLT and VT1 (*y*‐axis) for each individual against the mean of sLT and VT1 (*x*‐axis). (a) sLT vs. VT1 (s). (b) sLT vs. VT1 (w). SD: Standard deviation, sLT: Sweat lactate threshold, VT1: Ventilatory threshold.

A strong correlation was observed between sLT and VT1 (Figure 3). The correlation was stronger when expressed in terms of workload than in terms of exercise time (Figure 3a: sLT [s] vs. VT1 [s]: *r* = 0.859, *p* < 0.001; Figure 3b: sLT [W] vs. VT1 [W]: *r* = 0.904, *p* < 0.001). Bland–Altman analysis revealed mean differences of −7.7 s and 0.2 W between the thresholds (Figure 4), respectively. No fixed bias was observed, as the mean differences and their 95% confidence intervals included zero (sLT [s] vs. VT1 [s]: *p* = 0.419, 95% confidence interval: −27.1138–11.6323; sLT [W] vs. VT1 [W]: *p* = 0.832, 95% confidence interval: −1.5908–1.9612). In addition, no proportional bias was detected, as regression analysis showed no significant relationship between the differences and means (sLT [s] vs. VT1 [s]: *r* = 0.201, *p* = 0.315; sLT [W] vs. VT1 [W]: *r* = 0.195, *p* = 0.331). These findings indicate strong agreement between sLT and VT1.

Table 2 shows the changes in heart rate, VO_2_, sweat rate, and sweat lactate during the arm crank exercise test. Heart rate, VO_2_, and sweat rate increased during warm‐up and as load increased, but no significant differences were observed between the values at sLT and VT1. In contrast, the sweat lactate concentration did not differ significantly at the warm‐up, sLT, or VT1 time points, whereas a significant increase was observed only at the end of exercise.

**TABLE 2:** Changes in heart rate, VO_2_, sweat rate, and sweat lactate during incremental upper limb exercise.

	Warm‐up onset	End of warm‐up	sLT point	VT1 point	End of exercise
Load [W]	0	M:20, F:10	23.0	22.8	35.0
	(0)	(0)	(10.5)	(9.6)	(12.5)
Heart rate [bpm]	72.7^a^, ^b^, ^c^, ^d^	95.1^d^, ^e^	109.9^d^, ^e^	112.6^d^, ^e^	143.2
	(14.4)	(16.8)	(18.2)	(15.9)	(15.4)
VO_2_ [mL min^−1^ kg^−1^]	4.0^a^, ^b^, ^c^, ^d^	10.1^b^, ^c^, ^d^, ^e^	11.7^a^, ^d^, ^e^	11.9^a^, ^d^, ^e^	16.1
	(0.6)	(1.3)	(2.7)	(2.8)	(3.9)
Sweat rate [mg cm^−2^ min^−1^]	0.00^a^, ^b^, ^c^, ^d^	0.07^d^, ^e^	0.09^d^, ^e^	0.19^d^, ^e^	0.59
	(0.04)	(0.11)	(0.16)	(0.29)	(0.61)
Sweat lactate [μA]	4.12^d^	4.11^d^	4.05^d^	4.54^d^	5.71
	(0.98)	(0.98)	(1.22)	(1.66)	(2.13)

*Note*: Data are presented as the mean (standard deviation).

^a^ Versus end of warm‐up (*p* < 0.05).

^b^ Versus sLT point (*p* < 0.05).

^c^ Versus VT1 point (*p* < 0.05).

^d^ Versus end of exercise (*p* < 0.05).

^e^ Versus warm‐up onset (*p* < 0.05).

## 4 DISCUSSION

The main finding of this study was that the AeT identified using a sweat lactate sensor during arm crank ergometer exercise closely corresponded with VT1, which is widely regarded as the gold standard for AeT determination. Strong correlations were observed between sLT and VT1 at both exercise time and workload, and the Bland–Altman analysis demonstrated no fixed or proportional bias. These results support our hypothesis that sweat‐based AeT assessment is feasible during incremental upper limb exercise, extending previous findings obtained during lower limb exercise testing.

Previous studies, including those comparing upper and lower limb exercise, have suggested that although the absolute values of aerobic or anaerobic thresholds differ depending on the exercise modality, the threshold concept itself may be preserved across different forms of exercise. Orr et al. reported that the anaerobic threshold estimated from expired gas analysis during arm crank exercise occurred at a significantly lower oxygen uptake than that during cycling; nevertheless, a moderate correlation was observed between the two thresholds (Orr et al., 2013). Importantly, upper limb exercise is associated with lower peak oxygen uptake and earlier anaerobic threshold compared with lower limb exercise (Davis et al., 1976; Muraki et al., 2004; Orr et al., 2013; Reybrouck et al., 1975). Despite these modality‐specific differences, the present results indicate that the temporal coupling between systemic ventilatory responses and sweat lactate dynamics is preserved. This may reflect the fact that once systemic lactate production begins to rise in association with increasing metabolic demand, sweat lactate concentrations increase in parallel, allowing the sweat‐derived breakpoint to track the underlying metabolic transition even at lower absolute workloads. In addition, it should be noted that threshold responses may also be influenced by the duration of each exercise stage because physiological responses during incremental exercise exhibit temporal dynamics. Therefore, the present findings should be interpreted within the context of the specific step increment protocol employed in this study.

Environmental conditions and measurement media may also influence physiological responses during exercise. We previously investigated the validity of AeT assessment during swimming under aquatic conditions and demonstrated a significant correlation between swimming velocity at sLT and the velocity corresponding to LT1 estimated from blood lactate concentration (Okawara et al., 2024). These findings indicate that sweat lactate–based threshold detection in sweat can reflect established gold‐standard indices, such as VT1 or LT1, even under markedly different exercise environments. Taken together, these previous findings support the notion that, in individuals who exhibit sufficient sweating, physiological thresholds derived from ventilatory or blood lactate measurements can be captured using alternative noninvasive approaches across different exercise modalities. The present study demonstrated close agreement between sLT and VT1 during incremental upper limb exercise, supporting the validity of the sweat‐based AeT assessment.

In the present study, the onset of sweating generally preceded sLT, indicating that sLT does not simply reflect the initiation of sweating. Although both sweat lactate and sweat rate increased with exercise intensity, the temporal dissociation between the onset of sweating and sLT suggests that sLT represents a metabolic transition accompanying exercise rather than reflecting the onset threshold for sweating driven by increases in core and skin temperatures. This contrasts with our previous findings in healthy women, in which the delayed onset of sweating was associated with a systematic delay of sLT relative to VT1 (Sawada et al., 2026). In this population, sweating dynamics significantly influenced the discrepancy between the two thresholds, particularly in individuals with a late onset of sweating. In the present cohort, sweating generally preceded or occurred close to sLT in most participants, likely reflecting the inclusion of healthy participants with intact sweating responses. Under such conditions, sLT closely corresponded to VT1 without fixed or proportional bias. These findings support the interpretation that sLT reflects a dynamic metabolic breakpoint once sufficient sweating is established, rather than merely indicating the onset or magnitude of sweat production. Nevertheless, delayed sweating may reduce the accuracy of sLT detection in some individuals. Consistent with our previous findings (Sawada et al., 2026), sufficient sweat production appears to be a prerequisite for reliable threshold detection. Therefore, practical strategies that promote sweating, such as an adequate warm‐up and hydration, may be important when applying this method in real‐world settings.

The heart rate increased progressively throughout the exercise test, with no significant differences between the values at the sLT and VT1. This further supports the physiological correspondence between these thresholds and reinforces the validity of the sweat lactate–based AeT assessment during upper limb exercise. Owing to its noninvasive nature and capacity for continuous monitoring, this approach may provide a practical alternative to conventional respiratory gas analysis or blood lactate measurements. Unlike laboratory‐based assessments that require specialized equipment, sweat lactate monitoring has the potential to facilitate individualized exercise intensity prescriptions in more accessible settings. Importantly, the present findings also demonstrate that AeT can be evaluated during upper limb exercise, suggesting its potential applicability in populations for whom lower limb exercise is limited. Although individuals with impaired thermoregulatory function, such as those with high‐level spinal cord injury, may present altered sweating responses, the present study indicates that when sufficient sweating is preserved, sweat lactate monitoring can reflect metabolic thresholds during exercise. Further studies are warranted to determine the feasibility of this method in clinical populations with altered autonomic or sudomotor function.

This study has several limitations should be carefully considered. First, the participants were healthy young adults, and the applicability of these findings to older individuals and clinical populations remains to be established. Second, sweat lactate was measured only at the forehead under controlled environmental conditions, and sweating response may vary depending on thermal and environmental factors. Therefore, the generalizability of the present findings to other measurement sites or environmental conditions remains to be determined. Third, although longer stage durations (e.g., 3 min or longer) are generally recommended for assessing blood lactate thresholds, the 1‐min step incremental protocol used in this study may have been too short to fully reflect changes in blood lactate concentration in response to increasing exercise intensity. However, because sweat lactate is considered to reflect local sweat gland metabolism rather than blood lactate concentration directly, a shorter incremental protocol may still be appropriate for detecting the sweat lactate inflection point while reducing test duration and participant burden. Fourth, maximal aerobic capacity was not formally assessed in the present study. The exercise protocol was designed to identify the sLT based on the inflection point of continuously measured sweat lactate during incremental exercise, rather than to evaluate maximal aerobic capacity. Therefore, participants were not required to exercise until volitional exhaustion, and some terminated the test before reaching their maximal effort once sufficient data were obtained to determine the sLT. While this approach reduces excessive physical burden and enhances the feasibility of AeT assessment, it limits interpretation of the results in relation to maximal aerobic capacity. Future studies should investigate the validity of sweat‐based AeT assessment in populations with limited lower limb function, such as older adults, individuals with spinal cord injury, or those with lower limb joint disorders, as well as under a wider range of exercise and environmental conditions.

## 5 CONCLUSION

AeT identified using a sweat lactate sensor during arm crank exercise closely corresponds to VT1, with no fixed or proportional bias. These findings demonstrate that sweat lactate dynamics can serve as a noninvasive marker of metabolic transition even during upper limb exercise, supporting the robustness of sweat‐based AeT assessment across exercise modalities.

## AUTHOR CONTRIBUTIONS

**Tomonori Sawada:** Conceptualization; data curation; formal analysis; funding acquisition; investigation; methodology; software; validation; visualization. **Hiroki Okawara:** Conceptualization; data curation; formal analysis; funding acquisition; investigation; methodology; software; validation; visualization. **Kazuki Minami:** Data curation; formal analysis; investigation. **Shintaro Narushima:** Data curation; formal analysis; investigation. **Ayaka Shiratori:** Data curation; formal analysis; investigation. **Yoshinori Katsumata:** Resources; supervision; validation. **Masaya Nakamura:** Funding acquisition; resources; supervision. **Takeo Nagura:** Funding acquisition; project administration; resources. **Daisuke Nakashima:** Conceptualization; funding acquisition; methodology; project administration; resources; validation; visualization.

## FUNDING INFORMATION

This study was supported by JSPS KAKENHI (grant number: JP22K11477), General Insurance Association of Japan, National Mutual Insurance Federation of Agricultural Cooperatives (Zenkyoren), and Hisamitsu Pharmaceutical Co., Inc.

## CONFLICT OF INTEREST STATEMENT

Daisuke Nakashima is the president of Grace imaging Inc. and holds shares in the company. This company was not involved in the study. The remaining authors declare no competing financial interests or personal relationships that could influence the work reported in this study.

## ETHICS STATEMENT

This study was approved by the Institutional Review Board of Keio University School of Medicine (approval number: 20180357).

## CONSENT STATEMENT

All participants provided written informed consent before participating in this study.

## Data Availability

The data supporting the findings of this study are available from the corresponding author upon reasonable requests.
